# Prostate Cancer Metastasis to Stomach: A Case Report and Review of Literature

**DOI:** 10.3390/curroncol30040295

**Published:** 2023-03-30

**Authors:** Leena Moshref, Mohammad Abidullah, Piotr Czaykowski, Amitava Chowdhury, Robert Wightman, Pamela Hebbard

**Affiliations:** 1 Department of Surgery, University of Manitoba, Winnipeg, MB R3E 0V9, Canada; 2 Department of Pathology, University of Manitoba, Winnipeg, MB R3E 3P5, Canada; 3 Section of Hematology/Oncology, Department of Internal Medicine, University of Manitoba and CancerCare Manitoba, Winnipeg, MB R3E 0V9, Canada; 4 Department of Radiation Oncology, CancerCare Manitoba, Winnipeg, MB R3E 0V9, Canada; 5 Department of Surgical Oncology, CancerCare Manitoba, Winnipeg, MB R3E 0V9, Canada

**Keywords:** prostate-specific antigen (PSA), gastrointestinal tract (GI tract), immunohistochemistry (IHC)

## Abstract

Metastatic prostate cancer is a common diagnosis with a protracted but terminal course. Gastrointestinal (GI) tract involvement is extremely rare, and reportedly portends a poor prognosis. It can present years after the initial prostate cancer diagnosis. Only fifteen cases of prostate cancer metastasis to the stomach have been reported in the literature. We report a case of a 72-year-old man with metastatic castration-resistant prostate cancer and extensive bony involvement. He presented a decade after the diagnosis of prostate cancer with signs of heartburn; a gastric biopsy was initially believed to represent primary gastric carcinoma, but subsequently a diagnosis of prostate cancer metastatic to the stomach was confirmed. This case highlights the importance of the provision of a pertinent clinical history and clinical differential diagnosis at the time of submission of surgical pathology specimens, as well as highlighting the need to have a low index of suspicion to pursue additional pathologic markers whenever a presumed second adenocarcinoma is noted in the context of a patient having a history of current or prior advanced-stage adenocarcinoma of another site. The correct diagnosis can shield the patient from the morbidity of inappropriate surgical or medical management.

## 1. Introduction

Prostate cancer is the most common malignancy in Canadian males [1], but only the third most common cause of cancer-related death in men. The 5-year net survival is excellent, at ~91% [2]. Although only ~10% of men diagnosed with prostate cancer have Stage IV (metastatic) disease at diagnosis, a significant proportion of men with initially early-stage disease will eventually develop metastases. The 5-year net survival for men with metastatic prostate cancer is ~43% [3]. The bones and lymph nodes are by far the most common sites of distant metastasis in prostate cancer, with the lungs and liver much less commonly affected [4]. The “dependency of the seed on fertile soil” theory has historically been used to explain the organ-specific pattern of prostate cancer spread [5]. According to this theory, cancer cells will metastasize with a high affinity to certain areas with a favorable tumor microenvironment. This presumption has led to the traditional view that the lymph nodes and bone are the “fertile soils” for prostate cancer. The importance of epithelial-to-mesenchymal transition (EMT) as a driver of invasion and metastasis has been recognized more and more recently as there is evidence of a link between EMT and the cancer stem cells that initiate and maintain tumors and may be involved in invasion and metastasis. However, a more contemporary view of this hypothesis is that cancer cells carry their own soil from the primary site in the form of stromal components, including activated fibroblasts [5].

Notably, gastrointestinal (GI) tract involvement is extremely rare. Only a few cases of prostate cancer metastasis to the GI tract, including the small bowel [6], esophagus [7,8], and stomach [4,9,10,11,12,13,14,15,16,17,18,19,20,21,22], have been reported. The path of metastasis to the GI tract is unknown, although lymphatic channels have been proposed [23]. Cancer metastatic to the stomach from any solid tumor is uncommon, with a 5.4% incidence rate reported among 6380 autopsy cases [24]; in this case series, the most common solid tumors metastasizing to the stomach were malignant melanoma, followed by breast, esophageal, and lung cancer; only two cases (2.5%) of prostate cancer were identified [24].

Furthermore, prostate cancer can cause peritoneal metastasis without bone or lymph node metastasis; however, the pathological mechanism remains unclear, but it may be related to certain histological subtypes of prostate cancer with unusual biological behavior and/or particular patterns of metastatic spread, such as mucinous adenocarcinoma [5].

We report the first case of prostate cancer metastatic to the stomach in Canada. In addition, this paper summarizes all previous case reports of prostate cancer metastasis to the stomach.

## 2. Case Description

### 2.1. Materials and Methods

The current study was a case report and a review of the literature. The case report was gathered from electronic records. A review of the literature was done using a PubMed search engine. Out of 568 searches in PubMed, only 20 papers were relevant to our paper. Studies published in English were included (a total of fifteen studies) and have been summarized. Consent has been obtained from the patient for the publication of this study.

### 2.2. Case Report

The patient in question was diagnosed at age 65 with high-volume, high-grade (Gleason score 5 + 4 = 9) adenocarcinoma of the prostate. PSA was 9.6 ng/mL. No metastases were identified on staging investigations. He received 3 months of neoadjuvant androgen deprivation therapy with an LHRH agonist, followed by a radical prostatectomy. The final surgical pathology was pT3bN0 Gleason 4 + 5 invasive prostatic adenocarcinoma, acinar (usual) type. The surgical margins were clear but there was bilateral seminal vesicle involvement.

He had a family history of cancer. It included a brother with bladder cancer and another brother with prostate cancer. In addition, one of his daughters died her in 20s of a brain tumor.

The PSA was never unmeasurable post-prostatectomy. He declined salvage radiotherapy.

Approximately 1-year post-surgery, he was noted to have a sacral metastasis and received 1 year of androgen deprivation therapy (ADT).

Four years later, with a significant rise in PSA (PSA 21.35 ng/mL), imaging confirmed the progression of bony metastatic disease, without nodal or visceral metastases. ADT was restarted with a transient decline in PSA. Six years from his initial diagnosis, enzalutamide was added, with an excellent PSA response down to 0.14 ng/mL.

After 9 months, the PSA started to rise again. Imaging confirmed progression in the bones, without non-bony metastases. He was offered standard chemotherapy with docetaxel and prednisone, but, since he was feeling well, he elected not to proceed with the chemotherapy. Enzalutamide was discontinued and he remained on an LHRH agonist alone. Despite serial rises in PSA, he elected not to make any change in his management. Imaging continued to demonstrate only bony metastases.

**Figure 1:**
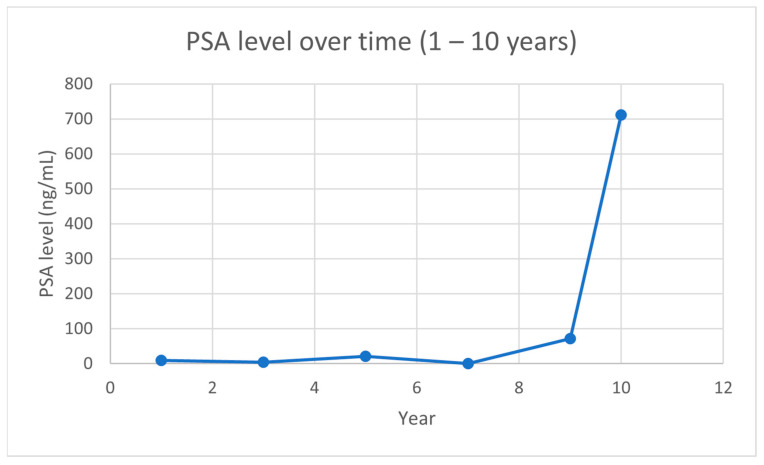
PSA level over time (1–10 years).

Approximately 1 year later (9 years from initial diagnosis), he presented with severe gastrointestinal reflux symptoms. It was noted that there had been a steady progression of anemia in the preceding months, and hemoglobin had dropped to ~100 g/L. PSA had risen to 712 ng/mL (Figure 1. Small pulmonary nodules were noted on CT chest imaging. There was no obvious gastric abnormality on CT imaging. He underwent upper and lower GI tract endoscopy. This identified an ulcerated area along the greater curvature of the stomach, suspicious for gastric cancer.

**Figure 2:**
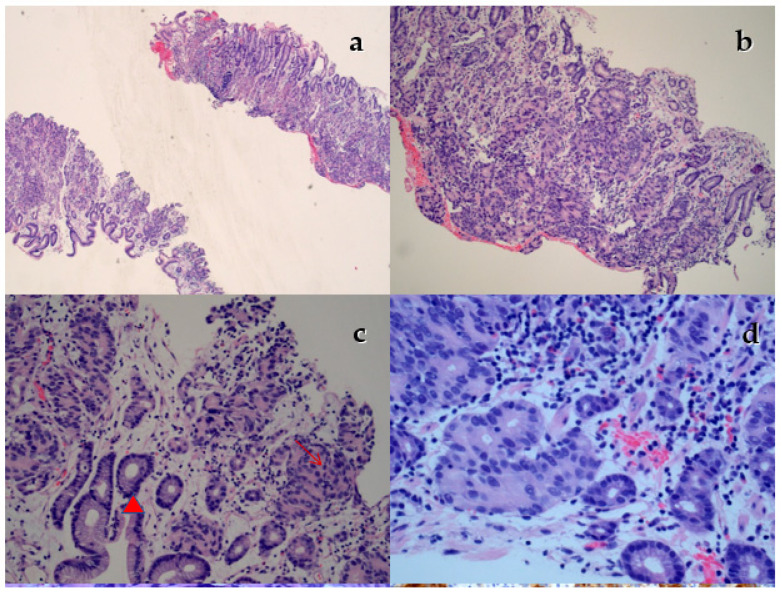

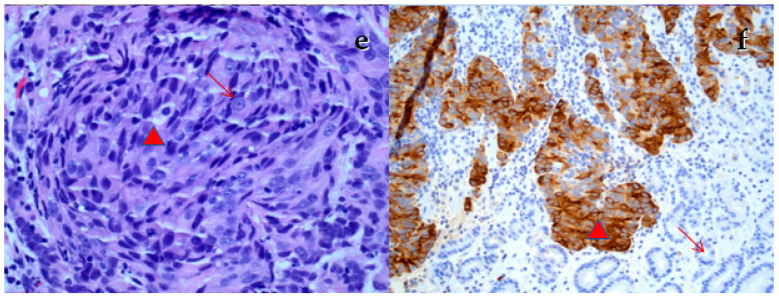
(**a**,**b**) show malignant glands within the lamina propria with overlying benign mucosa. (**c**) shows benign gastric mucosa on surface (arrowhead) and malignant glands (arrow). (**d**) High power of malignant glands. (**e**) shows solid areas of tumor with mitotic figures (arrowhead) and prominent nucleoli (arrow). (**f**) shows malignant glands with diffuse strong cytoplasmic staining with PSA (arrowhead) with negative staining in benign gastric mucosa (arrow).

A biopsy of the gastric mass was performed (Figure 2). The specimen was submitted with clinical history of “gastroscopy; gastric mass/cancer” and was labelled “Gastric Mass Bx”. The specimen consisted of 8 tissue fragments, ranging in size from 0.2 to 0.4 cm. No additional clinical history was provided at the time of submission of the specimen. Microscopic examination of the tissue fragments revealed benign gastric mucosa with an interspersed, distinct second population of carcinoma with a mixture of glandular differentiation and foci of solid sheets of malignant cells. The malignant glands were mitotically active with hyperchromatic nuclei, prominent nucleoli, and a high NC ratio. The case was signed out as invasive adenocarcinoma.

The case was reviewed at one of the multidisciplinary rounds and the possibility of a metastatic prostatic adenocarcinoma was discussed in the differential diagnosis. The case was referred back to the index pathologist, who performed immunohistochemical stains to confirm or exclude the possibility of a metastatic prostatic adenocarcinoma. The tumor cells showed diffuse strong cytoplasmic staining with prostate-specific antigen (PSA), with negative staining with CK 7, CK 20, and CDX2. The immunohistochemical profile of the tumor was consistent with metastatic prostatic adenocarcinoma and the initial report was accordingly amended.

Immunohistochemical stains are not routinely performed on primary tumors at sites where they occur on a regular basis. For instance, no immunohistochemical stains are routinely performed on colorectal carcinomas submitted with a clinical history of masses or cancers. On the other hand, immunohistochemical stains are routinely performed when there is clinical suspicion of a metastatic disease or unusual morphological features of a tumor at an unusual site. This case highlights the importance of the provision of a pertinent clinical history and clinical differential diagnosis at the time of submission of surgical pathology specimens.

With these results, the patient was offered palliative radiotherapy to the stomach, and he received a total of 20 Gy in 5 fractions to control gastric bleeding. He was once again offered systemic therapy but declined. He passed away after the last follow-up, around nine months after the diagnosis of the gastric metastasis, but with declining function and having been enrolled in palliative care.

## 3. Discussion

Gastric metastasis was found to occur in 1% to 4% of cases in two postmortem studies of 67 and 347 patients with metastatic prostate cancer, respectively [23,24]. There have only been 16 cases of metastatic gastric tumors of prostate cancer origin documented in the English literature [4,9,10,11,12,13,14,15,16,17,18,19,20,21,22]; these are summarized in Table 1. The usual modes of dissemination of cancer are contiguous infiltration or lymphatic, or hematogenous spread. Lymphatics may be used for metastases to the gastrointestinal tract since the prostate is well supplied with lymphatic channels [23].

It has been reported in the literature that gastric cancer can present as bone metastases without any preceding gastrointestinal symptoms [25]. Bone metastasis in gastric cancer patients is rare and carries a poor prognosis, with a mean survival time of 4–5 months [26]. When a primary malignancy has diffused stomach involvement; a Borrmann type 4 morphology, poorly differentiated adenocarcinoma, or signet ring cell carcinoma is present at an earlier age; and there are numerous nearby lymph node metastases, bone metastasis may be more common [26]. The cancer cells diffusely proliferate in the bone marrow during bone metastasis, which can result in disseminated carcinomatosis. They also multiply quickly, which results in bone damage and hematological problems. It is unknown how precisely tumor cells spread to the bone. The abundance of blood capillaries in the gastric mucosa was thought to play a role in the early dissemination of cancer to the bone. The vertebral venous plexus has also been proposed as a potential alternative non-portal pathway for bone metastasis from stomach malignancy [26].

The symptoms that have been more commonly described when prostate cancer has spread to the stomach include nausea and vomiting [4,9,10,11,12], abdominal pain [9,12,16,17,22], and anorexia [10,13]. Back discomfort, dysphagia, anasarca, hematemesis, melena, decreased appetite, and other symptoms have also been noted [9,10,11,12,13,18,19]. In our case, the patient presented with epigastric discomfort, heartburn, decreased appetite, and nausea. In addition, prostate cancer spread to the stomach can present as anemia [14,15].

At least one study has demonstrated that prostate cancer metastasizing to the stomach is a late event, usually taking around ten years from the diagnosis of advanced prostate cancer [20]. Most patients present with a high PSA of more than 150 ng/mL at the time of metastasis to the stomach [9,10,11,13,15,17], and some more than 1000 ng/mL [4,11,14,18,19]. However, two cases noted lower levels of PSA of less than 30 ng/mL [12,16], and one case had a normal PSA level of 0.152 ng/mL. Our study was congruent with the literature as there was a high PSA level of 467.8 ng/mL at the time of gastric metastasis.

In a case reported by Shindo of gastric metastases from prostate cancer, there was no elevation of PSA, but serum levels of CEA and CA19-9 were extremely high, indicating that these serum markers are not sufficiently specific to differentiate metastatic gastric tumor of prostate cancer origin from primary gastric cancer [20]. Hepatobiliary cancer and gastrointestinal cancer frequently have elevated tumor markers including CEA and CA19-9, but elevation of these markers is rare in prostate cancer. These markers’ unusually high levels in cancer can sometimes be a sign of poor differentiation. There are two forms of primary prostate cancer with selective metastatic spreading, according to Guthman et al. [27]. The CEA and PSA expression patterns of these two kinds were different. While CEA-positive/PSA-negative cancer cells were discovered in liver metastasis, PSA-positive/CEA-negative cancer cells metastasized to lymph nodes [28].

Regarding endoscopy, the most common finding is gastric ulceration [4,11,12,13]. Other endoscopic findings include a severely dilated stomach with extensive rugal folds, atypical/hypertrophic mucosal folds, pronounced mucosal convergence, friable or depressed lesions extending across the mucosa, and mucosal nodules [9,14,15,16,17,19,20,21,22].

On histopathology, metastatic prostatic adenocarcinomas can be difficult to differentiate from a primary carcinoma of another organ on standard H&E staining, and the role of clinical history and clinical differential diagnosis cannot be over-emphasized. Retrospectively, in our case report, the histopathology of gastric mucosa did not show intestinal metaplasia or dysplasia involving the surface mucosa, features expected to be seen in primary gastric carcinoma. However, it is not uncommon not to see these features, especially if the biopsy is taken from the edge of a lesion with the extension of malignant glands from the adjacent focus of invasive primary carcinoma. In such cases, primary invasive adenocarcinoma can be present in the lamina propria and submucosa, with no evidence of intestinal metaplasia or dysplasia in the overlying surface mucosa. As such, it would be difficult to differentiate a primary carcinoma from a metastatic carcinoma without immunohistochemical stains.

If clinically or morphologically suspected, immunohistochemical stains are required to determine the primary site of the tumor. Various immunohistochemical stains, including prostate-specific antigen (PSA), prostate specific acid phosphatase/prostatic acid phosphatase (PSAP/PAP), NKX3.1, P501S, prostate-specific membrane antigen (PSMA), androgen receptor (AR), and alpha methyacyl CoA racemase (AMACR), are available, which can help to differentiate prostatic adenocarcinoma from carcinomas from other organs. PSA and PSAP are the two most common immunohistochemical stains, with sensitivity of 85–90% and both with high specificity [29]. PSA can be negative in 7–13% of prostatic adenocarcinoma cases. PSAP/PAP can be negative in 5% of Gleason score 8–10 prostatic adenocarcinomas. Moreover, PSA and PSAP/PAP immunoreactivity decreases after androgen deprivation therapy [29]. NKX3.1 has high sensitivity of 95% and in general has high specificity [29]. It has recently been used as a useful prostatic carcinoma marker in cases with negative PSA or PSAP/PAP staining. A review of the literature indicates that two cases of metastatic prostatic adenocarcinoma to the stomach had negative PSA staining [17,20], indicating that the absence of such staining is insufficient to distinguish prostate cancer from other malignancies. Additionally, these cases imply that when the presence of metastatic cancer cannot be ruled out based on the patient’s medical history, it is crucial to undertake IHC of the preoperative biopsy sample with several markers.

Of interest, one of these cases [20] led to a preoperative diagnosis of primary gastric cancer due to negative PSA staining. To rule out prostate cancer metastases, a gastrectomy with lymph node dissection was undertaken. The IHC analyses of the resected tumor revealed that the tumor was positive for additional prostate cancer markers, including alpha-methyl acyl-coenzyme A racemase (AMACR), prostate-specific acid phosphatase (PSAP), and androgen receptor, suggesting that the tumor was a metastatic lesion of prostate cancer rather than a primary gastric cancer [20].

Regarding the staining of gastric tissue, we performed staining using CK 7, CK 20, and CDX2 markers for the following reasons. A negative CK7 favors the diagnosis of prostate carcinoma over a urothelial tumor [29], and it is a negative marker for prostate ductal carcinoma [14]. However, CK 7 and CK 20 are nonspecific, and both can be positive in prostate carcinoma [29]. In addition, it is reported in 94% of high-grade prostate carcinomas that having a negative CDX2 would differentiate high-grade prostate carcinoma versus small-cell prostate cancer.

Recently, a small proportion of prostate acinar adenocarcinomas have been found to contain CDX2, a marker of intestinal epithelial cell development that is expressed in most primary and metastatic colorectal adenocarcinomas [30]. Prostatic adenocarcinomas with intestinal differentiation had a higher rate of positivity; except for one research work, where a lymph node metastasis and a brain metastasis both exhibited weak (1+) positivity for CDX2, metastatic deposits of prostate cancer have all tested negative for this marker [30].

In the past, radiation therapy for primary prostate cancer was said to reduce the number of poorly developed glands and cause nuclear pyknosis that resembled poor differentiation [31,32]. According to a report, radiation therapy may also have impacted the differentiation of any remaining prostate cancer cells after prostatectomy, making it impossible to make a preoperative diagnosis based on the pathological analysis of the biopsy sample [20].

As is the case with prostate cancer metastatic to any site, chemotherapy and hormonal therapy were the main forms of treatment in previously described cases [9,10,11,12,13]. Castration-resistant cases were managed with chemotherapy, and in hormone-sensitive cases, total androgen blockade was used [15]. These treatments can result in a decrease in PSA levels and the clinical stability of the disease. Docetaxel and prednisone are the most commonly used chemotherapy regimen [12]. Among the hormonal treatments, LHRH agonists and non-steroidal anti-androgens have been primarily employed [10,11,12,13]. Radiation therapy was used in one case, according to a study [11]. Additionally, megestrol was administered to stimulate appetite, ondansetron was given to treat nausea and vomiting, and morphine was given to treat pain [10,11,13]. Although radiotherapy was not reported very often in the literature, we used palliative radiotherapy for symptom relief.

In the majority of reported cases, GI metastasis (GIm) from prostate cancer is treated mostly with supportive and palliative care, with the goal of increasing quality of life for patients, as well as symptom control in those who are already experiencing symptoms. A fast diagnosis of GIm, along with intensive examinations, is still preferred in patients with advanced disease, as it can occasionally lead to significant adjustments in the therapeutic strategy that may be able to improve the prognosis for the illness [5].

It is important to note that the rarity of gastric metastases from prostate cancer means that there has been no attempt to systematically evaluate the response to the available systemic therapy options. As such, any treatment prescribed is done so in an empirical fashion, based on the assumption that gastric metastases will respond in a similar fashion to any other metastatic site of disease.

Appropriate systemic therapy options for prostate cancer metastatic to the stomach would thus depend on the “state” of the cancer, and the prior therapies attempted. In patients with gastric metastases diagnosed in the castration-sensitive state, systemic therapy would revolve around the initiation of androgen deprivation, either pharmacological (LHRH agonist or antagonist) or surgical (bilateral orchiectomy), likely accompanied by the intensification of therapy with an androgen receptor signaling inhibitor (ARSI—available agents include apalutamide, enzalutamide, and abiraterone/prednisone), or with chemotherapy (docetaxel) [33] or a combination of docetaxel plus an ARSI agent [34]. In the context of existing castration resistance, treatment options would depend on therapies already attempted; in addition to ARSI agents and docetaxel, cabazitaxel is a proven second-line chemotherapeutic [35]. In the case of our patient, the gastric metastases were diagnosed while he was in the castration-resistant state, and already on indefinite LHRH agonist therapy plus enzalutamide. He repeatedly declined additional therapy, and in particular chemotherapy.

### Prognosis

Prostate cancer metastases to the stomach are said to have a poor prognosis. Overall survival from the diagnosis of a GI tract metastasis until death is typically short (5 to 14 months) [11,12,36]. In patients with lymph node metastases from prostate cancer, visceral metastases represent an independent predictor of a poor outcome in multivariate analysis [5]. The prognosis of metastatic prostate cancer with elevated CEA and aggressive behavior has been noted to be poor [26,37].

## 4. Conclusions

To conclude, prostate cancer metastasis to the gastrointestinal tract is very rare, and it is associated with a poor prognosis. It can present years after the initial prostate cancer diagnosis. Patients typically present with nonspecific symptoms. The most common symptoms are heartburn, abdominal discomfort, nausea, vomiting, a loss of appetite, weakness, and fatigue. Thus, it can be difficult to establish a diagnosis of prostate cancer metastatic to the stomach. Although most cases present with a high PSA level at the time of gastric metastasis, some cases have a non-elevated PSA level. Therefore, a high index of suspicion is required.

Immunohistochemical stains are not routinely performed on primary tumor biopsies at sites where they occur on a regular basis. For instance, no immunohistochemical stains are routinely performed on colorectal carcinomas submitted with a clinical history of masses or cancers. On the other hand, immunohistochemical stains are routinely performed when there is clinical suspicion of a metastatic disease or unusual morphological features of a tumor at an unusual site. This case highlights the importance of the provision of a pertinent clinical history and clinical differential diagnosis at the time of submission of surgical pathology specimens.

Ultimately, gastric biopsies establish the final diagnosis in most cases, and the role of the clinical history and immunohistochemical assessment can be critical. Until now, there has been no definitive treatment for prostate cancer metastases to the stomach; treatments pursued are those generally used for treating advanced prostate cancer.

Finally, we would encourage clinicians to have a low threshold for requesting additional pathologic markers upon seeing a presumed second adenocarcinoma in the setting of a patient with a history of concurrent or prior advanced-stage adenocarcinoma of another site. In this case, we were able save the patient from the morbidity of noncurative gastric surgery and not overall survival.

## Figures and Tables

**Table 1:** Published studies of prostate cancer metastasis to stomach.

Case	Reference	Age (Years)	Stage at the Initial Diagnosis of Prostate Cancer	Gleason’s Score	Stage at Gastric Metastasis	Initial Presentation of Gastric Metastasis	PSA Level at the Diagnosis of Gastric Metastasis (ng/mL)	Time of Gastric Metastasis from Initial Diagnosis (Months)	Endoscopic Findings	IHC Findings	Histology Subtype	Treatment Received for Gastric Metastasis
1	Mehrzad et al. [4]	71	Metastatic	NA	Metastatic (bone and lymph nodes)	Nausea and anorexia	2250	132 months (11 years)	Superficial ulcerations and a 5 mm nodule	CK AE1/AE3 (+), PSA (+), CK7 (−), CK20 (−), CDX2 (−)	Adenocarcinoma	Disease progression on chemotherapy
2	Holderman et al. [9]	88	NA	2 + 5 (7)	No other metastases	Postprandial vomiting and epigastric discomfort	800	96	Nodules with central depression, fold thickening	PSA (+), CK (+), Mucin (−)	Adenocarcinoma (poorly differentiated)	Naive
3	Christoph et al. [10]	67	Metastatic	NA	Metastatic (bone and lymph nodes)	Severe nausea, vomiting, and anorexia	171	Initial finding	NA	PSA (+)	Adenocarcinoma (poorly differentiated)	Naive
4	Onitilo et al. [11]	57	Early stage	5 + 4 (9)	Metastatic (bone and brain)	Hematemesis	240	15	A broad base ulcerated exophytic lesion	PSA (+), CK (+), CG (−)	Adenocarcinoma	Disease progression on endocrine therapy
5	Onitilo et al. [11]	89	Metastatic	NA	Metastatic (bone)	Nausea, vomiting, decreased appetite, and anorexia	1565	Initial finding	Fold thickening with dispensability, ulceration	PSA (+), CK (+), CG (−)	Adenocarcinoma	Naive
6	Hong et al. [12]	66	Locally advanced	5 + 4 (9)	Metastatic (bone and lymph node)	Nausea, vomiting, and abdominal discomfort	17.9	18	Small elevations with ulceration	PSA (+)	Adenocarcinoma (undifferentiated)	Disease progression on endocrine therapy
7	Bilici et al. [13]	69	Metastatic	3 + 4 (7)	Metastatic (bone)	UGB	244.8	48	Multiple ulceration in gastric body	PSA (+), PSAP (+), CK7 (−), CK20 (−)	Adenocarcinoma	Clinical remission with endocrine therapy
8	Soe et al. [14]	64	Metastatic	5 + 4 (9)	Concurrent metastatic (lymph node and rectum)	Anemia and melena	More than 1000	16	Fold thickening	PSA (+), AMACR (+)	Adenocarcinoma	Withdrawing chemotherapy
9	Patel et al. [15]	71	NA	NA	Concurrent metastasis (sigmoid)	Anemia	More than 150	More than 10 years	A nodule, ulcer, and multiple erosions and a sigmoid polyp	Gastric fundus and sigmoid: PSA (+)	Adenocarcinoma	Status post surgery and radiation therapy
10	Bhandari et al. [16]	58	NA	NA	Metastatic (bone)	Epigastric pain	22.4	8	A nodule with ulceration in gastric antrum	PSA (+), CK20 (+), CK7 (−)	Adenocarcinoma	Disease progression on endocrine therapy
11	Inagaki et al. [17]	75	Metastatic	Not available	No other metastases	Epigastric discomfort	238	Not available	Slightly depressed, discolored lesion	PSA (−), PSAP (+), CK7 (−), CK20 (−)	Adenocarcinoma (moderately to poorly differentiated)	Responding to endocrine therapy
12	Tavukcu et al. [18]	67	Late gastric metastasis of ductal prostate cancer	3 + 3 (6)	Metastatic (bone, lymph node, and peritoneum)	Ascites and vomiting	4565	46	Suspicious area in gastric fundus	AMACR) (+), CK7 (−)	Ductal adenocarcinoma (undifferentiated)	Disease progression and death on TAB and chemotherapy
13	Koop et al. [19]	51	Metastatic	NA	Metastatic (lung)	Coffee ground emesis and melena	>4500	84	Mucosal nodule in the gastric body 1 cm in diameter with active bleeding	PSA (+)	Adenocarcinoma (high grade)	Supportively with platelet transfusion, intravenous proton pump inhibitor, and desmopressin with control of bleeding
14	Shindo et al. [20]	60	Metastatic	NA	Metastatic (bone)	Incidental	0.152	120 months (10 years)	Irregular depressed lesion with a convergence of folds at the greater curvature of the upper gastric body	PSA (−) in upper GI scope so laparoscopic proximal gastrectomy with lymphadenectomy was performed and PSA (+) in surgical specimen	Adenocarcinoma (poorly differentiated)	Status post-surgery (laparoscopic proximal gastrectomy with D1 and lymphadenectomy) and chemotherapy
15	Koklu et al. [21]	65	Metastatic	NA	Metastatic (bone and lung)	Pneumonia, hematemesis, and melena	NA	NA	Numerous umbilicated nodules of various sizes (0.5 to 2 cm) on the gastric mucosa	PSA (+)	Adenocarcinoma	Supportively with red blood cell transfusions and died of septic shock and respiratory failure
16	Krones et al. [22]	69	Metastatic	Naive	Naive	Abdominal discomfort	Naive	Naive	Hypertrophic gastric fold arising from upper third of the stomach	PSA and androgen receptors (+)	Adenocarcinoma	Naive
17	Our case	72	Metastatic	9/12 Gleason 9	Metastatic (bone)	Epigastric discomfort heartburn, decreased appetite, nausea	467.8	113	Large ulcerating lesion on the greater curve	PSA (++), few cells CK7 (+), CDX2 (−), CK20 (−)	Adenocarcinoma	Palliative radiotherapy

TAB: total androgen blockage, IHC: immunohistochemistry, (+): positive, (−): negative, CK: cytokeratin, CG: chromogranin, PSA: prostate-specific antigen, PSAP: prostate-specific alkaline phosphatase, AMACR: alpha-methyl acyl-coenzyme A racemase.

## Data Availability

The data presented in this study are openly available.
